# Supplementary material for: Human stem cell–derived neurons and astrocytes to detect novel auto-reactive IgG response in immune-mediated neurological diseases
Source: Front Immunol. 2024 Jul 24;15:1419712. doi: 10.3389/fimmu.2024.1419712 (PMC11303155; doi:10.3389/fimmu.2024.1419712)
Supplement: Supplementary file 1 [file DataSheet_1.pdf]

Supplementary Material

1 Supplementary Figures and Tables

1.1 Supplementary Figures

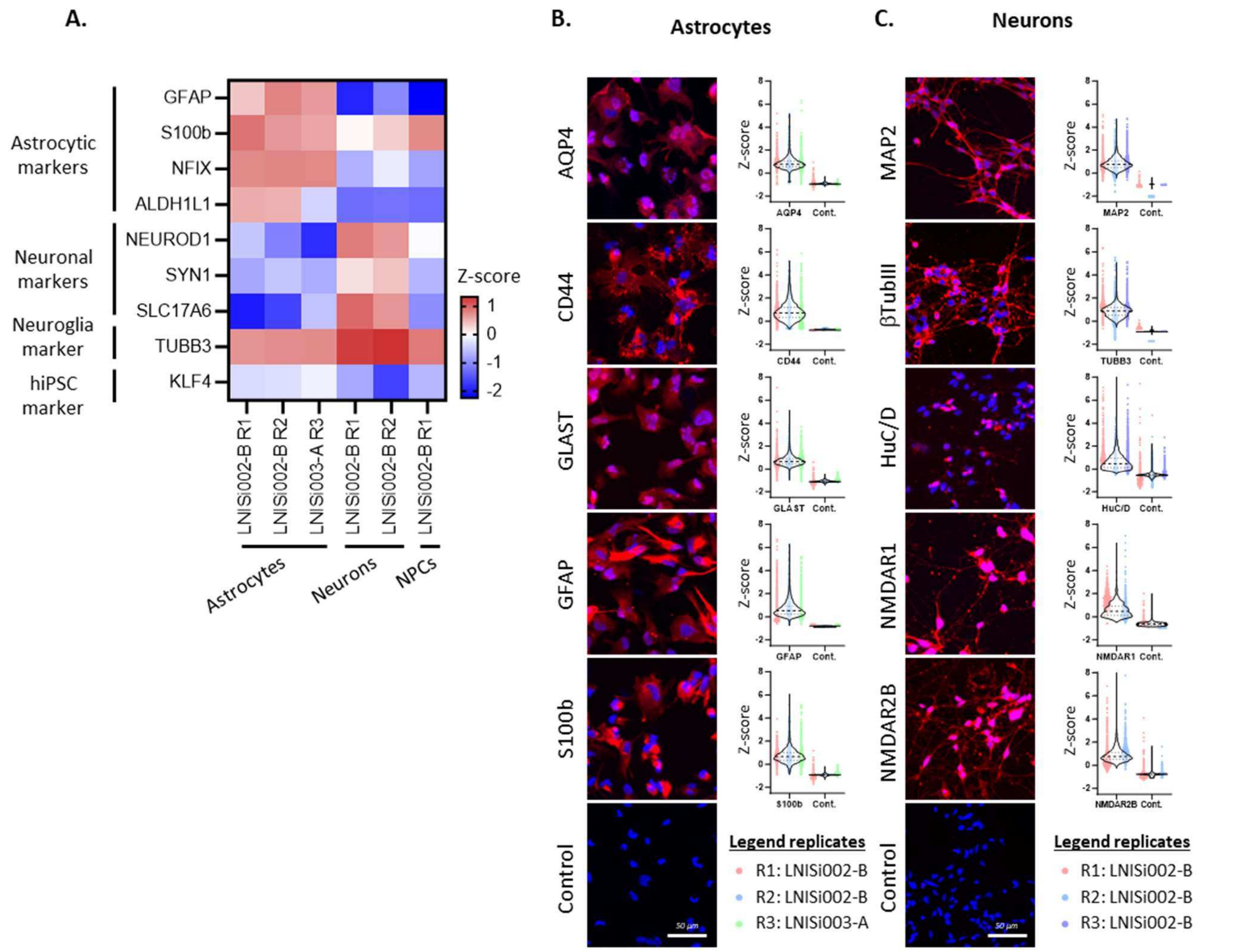

Supplementary Figure 1. Characterization of hiPSC-derived astrocytes and neurons used for the detection of CNS-reactive antibodies in the serum and CSF of selected patients.

(A) The expression profiles of nine astrocytic, neuronal and hiPSC markers are regularly evaluated in hiPSC-derived astrocytes, neurons and NPCs from healthy donors used in this study (HD#002 and HD#003). Representative experiments are reported for independent cultures (LNISi002-B, LNISi003-A). The nine markers are listed on the y axis and the cell type and donor are listed on the x-axis. Results are expressed as the *Z-score* of the -ΔC_T (C_T of gene of interest – C_T of GAPDH). **(B-C)** Specific astrocytic- (B; AQP4, CD44, GLAST, GFAP, S100β) or neuronal- (C; MAP2, β-Tubulin-III, HuC/D, NMDAR1 and NMDAR2B) marker expressions (red) were evaluated by immunofluorescence (IF). Nuclei stained in DAPI appear in blue. Control panels correspond to cells stained with secondary antibodies only. Scale bar, 50 µm. **Left panels:** Representative immunofluorescence images of hiPSC-derived astrocytes (B) or neurons (C) obtained from healthy control HD#002. **Right panels:** Selected marker expression was quantified at the single cell level using CellProfiler software (version 4.2.1). Each dot represents the mean fluorescence intensity of single cells in culture wells stained for selected markers and compared to control wells (Cont. : detection antibodies/reagents only; expressed as z-scores). A minimum of 10% (for neurons) / 25% (for astrocytes) of the total surface of the well were acquired to ensure representative cellular diversity (n=[12-20] single images to be analyzed; number of cells analyzed (median/interquartile range): astrocytes, 1234/1674; neurons, 2318/5907). Each color represents one differentiation from one donor (see legend on the figure). Each color represents one hiPSC-derived CNS cell differentiation from one donor (see legend on the figure for details).

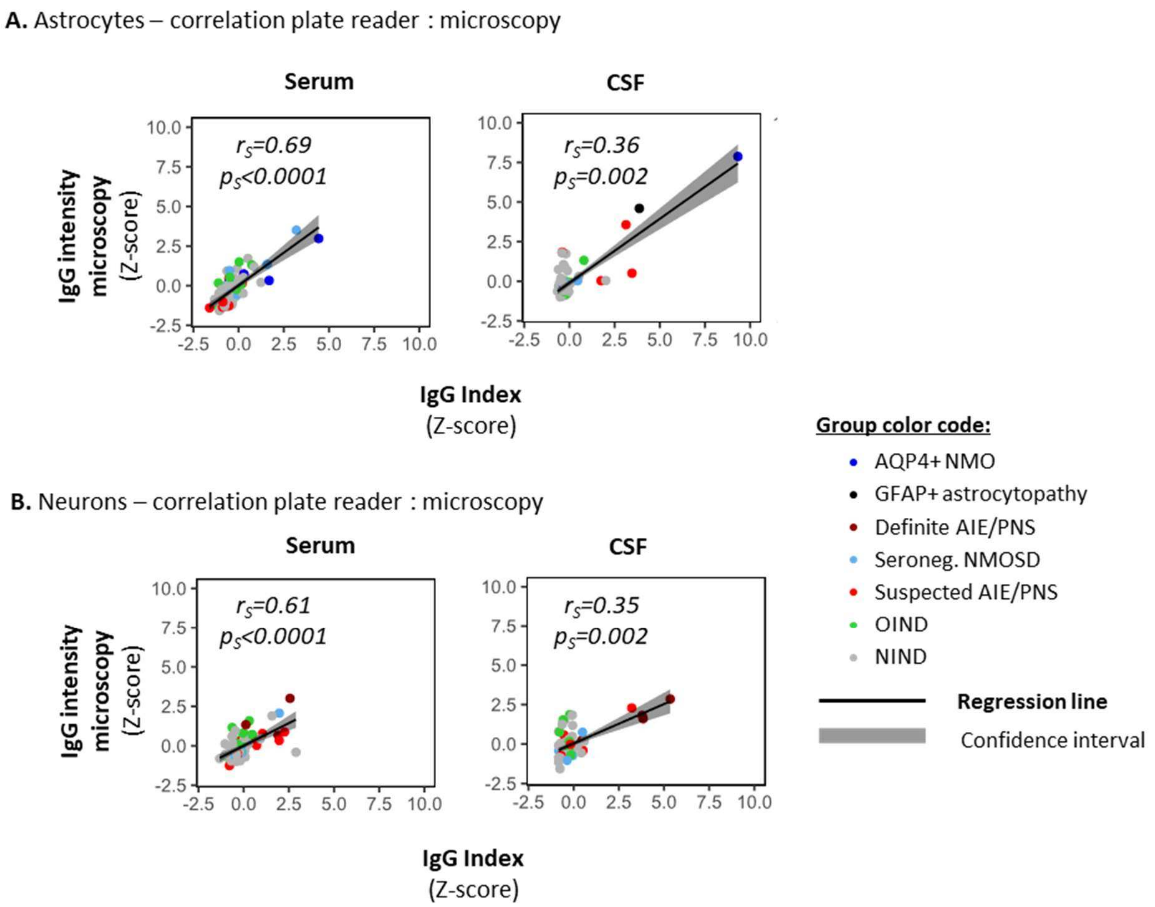

<u>Supplementary Figure 2.</u> Cross-validation of the automated plate reader rapid IgG measurement with single cell microscopy analyses.

Human iPSC-derived astrocytes **(A)** and neurons **(B)** were exposed to serum or CSF. FI were assessed using a Synergy® microplate reader. Subsequent IgG indexes (*z-score*) were calculated (see Methods for details). The exact same wells of the CBA were then observed by fluorescence microscopy using a EVOS M700 automated microscope plate reader and at least four images were acquired per well. Single-cell associated IgG intensity were then measured in all images. Correlations between IgG indexes at the well level (*z-score*) [x-axis] and the median IgG intensity at the single cell level by microscopy (*z-score*) [y-axis] were represented using a generalized linear model (reference line in plain black, confidence interval set at 95% in shadowed area) and tested using a Spearman's rank correlation test (r and p values on the graphs).

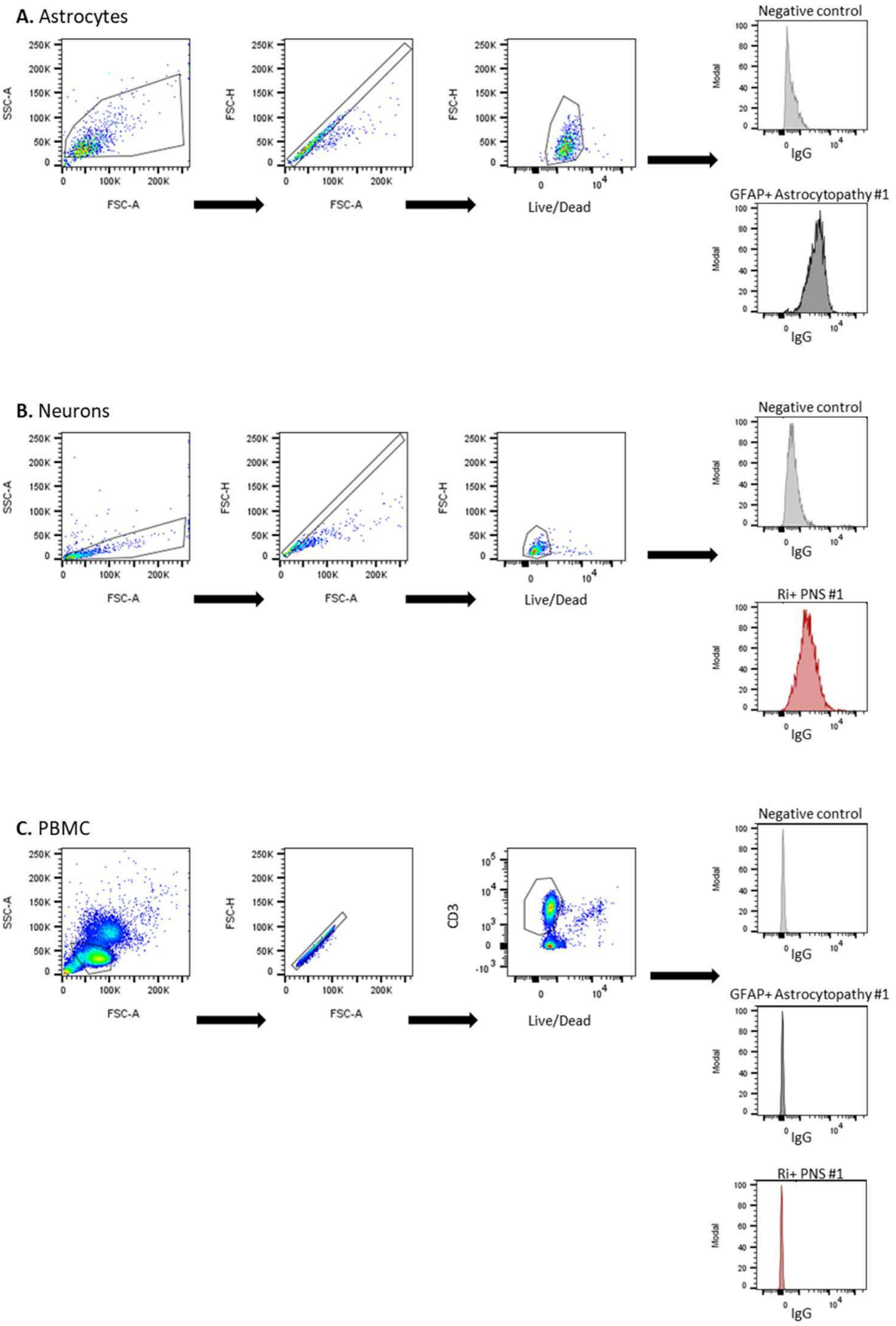

<u>Supplementary Figure 3.</u> **Gating strategy of hiPSC-derived astrocytes and neurons vs PBMC and subsequent analyses of IgG binding to CNS cells vs CD3+ T cells.**

Cells (**(A)** astrocytes, **(B)** neurons, **(C)** PBMC) were exposed to either IgG-detection antibodies only (Negative Control in grey), or selected patient CSF followed by IgG-detection Abs (A, C. GFAP+ astrocytopathy #1 in black, B-C. Ri+ PNS #1 in dark red). Total PBMCs were also stained using anti-CD3 antibodies. Dead cells were excluded using Live/dead marker. Representative gating strategies are represented. Data were acquired on LSRII flow and analyzed using Flow Jo Software.

1.2 Supplementary Tables

Supplementary Table 1. List of primers used for reverse transcription quantitative PCR (RT-qPCR).

GENE NAME	FORWARD	REVERSE
**GFAP**	GCCAGTTGCAGTCCTTGAC	GCGCATCTGCCTCTCCA
**NFIX**	CAAGGAGATGCGGACATCAAAC	ACCCCGGAAGTCACAAAACA
**S100B**	GCAGCAAGGAGACCAGGAA	CCACCATGGCCTTCTCCA
**KLF4**	CTGCGGCAAAACCTACACAA	CGTCCCAGTCACAGTGGTAA
**ALDH1L1**	GCTGACTGTGACCTCAACAA	GCTGCAATGCAATTCTCTCC
**NEUROD1**	GCCCCAGGGTTATGAGACTA	TCTGTCCAGCTTGGAGGAC
**SYN1**	GCAAGGACGGAAGGGATCA	TGTCTTCATCCTGGTGGTCAC
**TUBB3**	GAGCGGATCAGCGTCTACTA	GGTTCCAGGTCCACCAGAA
**SLC17A6**	TGGGGCTACATCATCACTCA	GAAGTATGGCAGCTCCGAAA

Supplementary Table 2. List of antibodies and detection reagents used for immunofluorescence assays.

Target	Clone	Manufacturer	Reference	Host	Dilution
Primary antibodies					
AQP4	polyclonal	Life Technologies	PA5-53234	rabbit	1/50
S100b	EP1576Y	Abcam	ab52642	rabbit	1/200
GLAST	polyclonal	Abcam	ab416	rabbit	1/200
GFAP	polyclonal	Millipore	Ab5804	rabbit	1/200
CD44	DB105	Miltenyi	130-113-333	biot	1/50
NMDAR1	polyclonal	Life Technologies	PA3-102	rabbit	1/50
NMDAR2B	polyclonal	Life Technologies	PA3-105	rabbit	1/50
btubIII	TuJ-1	biotechne	BAM1195	biot	1/100
HuC/HuD	16A11	Life Technologies	A-21271	mouse	1/50
MAP2	polyclonal	Abcam	AB5392	chicken	1/200
Detection antibodies/reagents					
anti-rabbit AF546	polyclonal	LIFE TECHNOLOGIES EUROPE BV	A10040	donkey	1/200
anti-mouse AF546	polyclonal	LIFE TECHNOLOGIES EUROPE BV	A10036	donkey	1/200
anti-chicken AF546	polyclonal	LIFE TECHNOLOGIES EUROPE BV	A11040	goat	1/200
anti-human IgG-biotin	poylclonal	Invitrogen	31774	goat	1/100
strepatvidin-PE	--	BD	554061	--	1/1000

Supplementary Table 3. Individual clinical information of the AQP4+ NMO patients enrolled in this study (part of validation cohort).

Subject	Year of diagnosis	Reference tests (Reference center) used at diagnosis	Immune modifying treatment[a]	Treatment duration[a]	Tested sample(s)[b]	Outlier 96-well CBA neuron[b]	Outlier 96-well CBA astrocyte[b]
AQP4+ NMO #1	2018	**IIFT monospecific CBA** (Sion): **AQP4**	N/A	--	SERUM CSF	n/a	**POS** (Serum/CSF)
AQP4+ NMO #2	2019	**IIFT monospecific CBA** (Geneva): **AQP4**	Rituximab	39 days	SERUM	n/a	**POS** (serum)
AQP4+ NMO #3	2005	**IIFT monospecific CBA** (Lyon): **AQP4**	Mycophenolate Mofetil	13.7 years	SERUM CSF	n/a	NEG
AQP4+ NMO #4	2012	**IIFT monospecific CBA** (Sion): **AQP4**	Mycophenolate Mofetil	7.1 years	SERUM	n/a	NEG

[a] Times expressed at sampling date

[b] Results from the 96-well hiPSC-derived CNS CBA are expressed as outlier **POS** (positive) or NEG (negative); n/a, not available.. If POS, in brackets the sample(s) to be tested as an outlier in the corresponding 96-well hiPSC-derived CNS CBA either using neurons or astrocytes.

Abbreviations: AQP4+ NMO, AQP4+ neuromyelitis optica; CBA, cell-based assay; IIFT, indirect immunofluorescence test; CSF, cerebrospinal fluid; N/A, not applicable

Supplementary Table 4. Individual clinical information of the Seronegative NMOSD patients enrolled in this study, who all were tested negative in reference laboratories (part of exploratory cohort).

Subject	Year of diagnosis	Reference tests (Reference center) used at diagnosis	Clinical manifestations	Immune modifying treatment[a]	Treatment duration[a]	Tested sample(s)[b]	96-well CBA neuron[b]	96-well CBA astrocyte[b]	Mouse TIF evaluation[c]
Seronegative NMOSD #1	2011	**IIFT monospecific CBA** (Lyon): AQP4	Severe bilateral ON and area postrema syndrome (hiccups)	N/A	--	SERUM CSF	**POS** (Serum/CSF)	**POS** (Serum/CSF)	NO
Seronegative NMOSD #2	2015	**IIFT monospecific CBA** (Lyon, Sion): AQP4, MOG	myelitis and ON	N/A	--	SERUM	NEG	**POS** (Serum)	NO
Seronegative NMOSD #3	2011	**IIFT monospecific CBA** (Lyon, Sion): AQP4, MOG	myelitis and optic neuritis	N/A	--	SERUM CSF	NEG	NEG	NO
Seronegative NMOSD #4	2021	**IIFT monospecific CBA** (Sion): AQP4, MOG	bilateral ON and myelitis	Azathioprin	53 days	SERUM CSF	NEG	NEG	NO
Seronegative NMOSD #5	2018	**IIFT monospecific CBA** (Sion): AQP4, MOG	severe ON and myelitis	Rituximab	0 days	SERUM	NEG	NEG	NO
Seronegative NMOSD #6	2021	**IIFT monospecific CBA** (Sion): AQP4, MOG	severe bilateral ON and myelitis	Azathioprin	2.3 years	SERUM CSF	NEG	NEG	NO

[a] Times expressed at sampling date

[b] Results from the 96-well hiPSC-derived CNS CBA are expressed as outlier **POS** (positive) or NEG (negative). If POS, in brackets the sample(s) to be tested as an outlier in the corresponding 96-well hiPSC-derived CNS CBA either using neurons or astrocytes.

[c] Samples from patients found to be positive in our novel 96-well hiPSC-derived CNS CBA were evaluated by indirect immunofluorescence on a mouse tissue composite (TIF) (YES or NO). If YES, in brackets the result of the mouse TIF.

Abbreviations: NMOSD : neuromyelitis optica spectrum disorder; ON, optic nerve; CBA, cell-based assay; IIFT, indirect immunofluorescence test; CSF, cerebrospinal fluid; N/A, not applicable.

<u>Supplementary Table 5.</u> Individual clinical information of the definite AIE/PNS patients enrolled in this study (part of validation cohort).

Subject	Year of diagnosis	Reference tests (Reference center) used at diagnosis	Immune modifying treatment[a]	Treatment duration[a]	Tested sample(s)[b]	96-well CBA neuron[b]	96-well CBA astrocyte[b]
anti-AK5+ AIE/PNS #1	2020	**IIFT mouse brain tissue** (Krone)<br>**IIFT monospecific CBA (Krone**): GAD-65, NMDAR, GABAAR, GABABR, IgLON5, AMPAR2, DPPX, LGI-1, CASPR2, Gly-R, mGluR5, mGluR1<br>**IIFT monospecific CBA (Barcelona): <u>AK-5</u>**<br>**Immunoblot** (Krone): Amphiphysin, CV2/CRMP5, Ma2, Ri, Yo, Hu, Recoverin, Sox1, Titin, Zic4, DNER	N/A	--	SERUM	NEG	n/a
anti-Hu+ AIE/PNS #1	2016	**IIFT mouse brain tissue** (Krone)<br>**IIFT monospecific CBA** (Krone): GAD-65, NMDAR, GABABR, AMPAR1, AMPAR2, DPPX, LGI-1, CASPR2, Gly-R, mGluR5<br>**Immunoblot** (Krone): Amphiphysin, CV2/CRMP5, Ma2, Ri, Yo, **<u>Hu</u>**, Recoverin, Sox1, Titin, Zic4, DNER	Methylpredni solone	12 days	SERUM CSF	**POS** (Serum/CSF)	n/a
anti-Hu+ AIE/PNS #2	2005	**Immunoblot** (Bern): **Hu**, Ri, Yo, Amphiphysin	N/A	--	CSF	**POS** (CSF)	n/a
anti-NMDA-R+ AIE/PNS #1	2013	**IIFT mouse brain tissue** (Bethel)<br>**IIFT monospecific CBA** (Bethel): GAD-65, GAD-67, **<u>NMDAR</u>**, GABABR, AMPAR, AQP4, LGI-1, CASPR2, Gly-R<br>**Immunoblot** (Bethel): Amphiphysin, CV2, Ma2, Ri, Yo, Hu	N/A	--	SERUM CSF	NEG	n/a
anti-PCA-Tr+ AIE/PNS #1	2015	**IIFT mouse brain tissue** (Bethel)<br>**IIFT monospecific CBA** (Bethel): GAD-65, NMDAR, GABABR, AMPAR1, AMPAR2, DPPX, LGI-1, CASPR2, Gly-R, mGluR5<br>**Immunoblot** (Bethel): Amphiphysin, CV2/CRMP5, Ma2, Ri, Yo, Hu, Recoverin, Sox1, Titin, Zic4, **<u>DNER/Tr</u>**	N/A	--	SERUM	NEG	n/a
anti-Ri+ AIE/PNS #1	2022	**IIFT primate brain tissue** (Sion)	N/A	--	SERUM CSF	**POS** (Serum/CSF)	n/a

		**Immunoblot** (Sion): GAD-65, Amphiphysin, CV2, Ma2, **Ri**, Yo, Hu, Recoverin, Sox1, Titin, Zic4, DNER/Tr					
anti-GFAP+ astrocytopathy #1	2020	**IIFT primate brain tissue** (Sion)<br>**IFT mouse brain tissue** (Krone)<br>**IIFT monospecific CBA** (Sion): NMDAR, GABABR, AMPAR1, AMPAR2, DPPX, LGI-1, CASPR2, Gly-R, mGluR5, IgLON5<br>**IIFT monospecific CBA** (Krone): **GFAP**, AQP4<br>**Immunoblot** (Sion): GAD-65, Amphiphysin, CV2/CRMP5, Ma2, Ri, Yo, Hu, Recoverin, Sox1, Titin, Zic4, DNER/Tr	N/A	--	CSF	n'a	**POS** (CSF)

[a] Times expressed at sampling date

[b] Results from the 96-well hiPSC-derived CNS CBA are expressed as outlier **POS** (positive) or NEG (negative) or n/a, not available. If POS, in brackets the sample(s) to be tested as an outlier in the corresponding 96-well hiPSC-derived CNS CBA either using neurons or astrocytes. Abbreviations: AIE : autoimmune encephalitis; PNS : paraneoplastic syndrome; CBA, cell-based assay; IIFT, indirect immunofluorescence test; CSF, cerebrospinal fluid; N/A, not applicable; n/a not available

Supplementary Table 6. Individual clinical information of the Suspected AIE/PNS patients enrolled in this study, who all were tested negative in reference laboratories (part of exploratory cohort).

Subject	Year of diagnosis	Reference tests (Reference center) used at diagnosis	Clinical manifestations	Tumor/ oncologic condition	Immune modifying treatment[a]	Treatment duration[a]	Tested sample(s)[b]	96-well CBA neuron[b]	96-well CBA astrocyte[b]	Mouse TIF evaluation[c]
Suspected AIE/PNS #1	2009	(Oxford): n/a[d]	opsoclonus myoclonus	N/A	N/A	--	CSF	NEG	**POS** (CSF)	**YES** (neg)
Suspected AIE/PNS #2	2021	**IIFT primate brain tissue** (Sion)<br>**IIFT monospecific CBA** (Sion): NMDAR, GABABR, AMPAR1, AMPAR2, DPPX, LGI-1, CASPR2, Gly-R, mGluR5, IgLON5<br>**Immunoblot** (Sion): G+V3:V13AD-65, Amphiphysin, CV2/CRMP5, Ma2, Ri, Yo, Hu, Recoverin, Sox1, Titin, Zic4, DNER/Tr	limbic encephalitis	N/A	N/A	--	SERUM CSF	**POS** (Serum)	NEG	NO
Suspected AIE/PNS #3	2018	**IIFT mouse brain tissu** (Krone)<br>**IIFT monospecific CBA** (Krone): GAD-65, NMDAR, GABABR, AMPAR1, AMPAR2, DPPX, LGI-1, CASPR2, Gly-R, IgLON5 mGluR5<br>**Immunoblot** (Krone): Amphiphysin, CV2/CRMP5, Ma2, Ri, Yo, Hu, Recoverin, Sox1, Titin, Zic4, DNER	limbic encephalitis	kidney tumor	N/A	--	SERUM CSF	NEG	NEG	NO
Suspected AIE/PNS #4	2012	**IIFT monospecific CBA** (Oxford): AQP4 CBA<br>**Immunoblot** (Zurich): Hu, Ri, Yo, Amphipysin, CV2, Ma2, Recoverin	encephalomyelitis	myeloid leukemia	N/A	--	CSF	**POS** (CSF)	NEG	NO
Suspected AIE/PNS #5	2008	None[d]	encephalomyelitis	B cell lymphoma	IFN-b1a	14 days	CSF	NEG	**POS** (CSF)	**YES** (neg)

Suspected AIE/PNS #6	2011	**Immunoblot** (Basel):GM1, GM2, GD1a, GD1b, GQ1b	Myasthenia gravis	thymoma	N/A	--	SERUM	NEG	NEG	NO
Suspected AIE/PNS #7	2019	**IIFT primate brain tissue** (Sion)<br>**IIFT monospecific CBA** (Sion): ABABR, AMPAR1, AMPAR2, DPPX, LGI-1, CASPR2, Gly-R, mGluR5, IgLON5<br>**Immunoblot** (Sion): GAD-65, Amphiphysin, CV2/CRMP5, Ma2, Ri, Yo, Hu, Recoverin, Sox1, Titin, Zic4, DNER/Tr<br>**Immunoblot** (Geneva): GM1, GM2, GD1A, GD1B, GQ1b<br>**Immunoblot** (Krone): Musk	post-ICI oculomotor nerves myositis	melanoma with lung metastasis	pembrolizumab --> ipilimumab --> nivolumumab	3.3 months	SERUM	NEG	NEG	NO
Suspected AIE/PNS #8	2018	**IIFT mouse brain tissue** (Krone)<br>**IIFT monospecific CBA** (Krone): GAD-65, NMDAR, GABABR, AMPAR1, AMPAR2, DPPX, LGI-1, CASPR2, Gly-R, mGluR5<br>**Immunoblot** (Krone): Amphiphysin, CV2/CRMP5, Ma2, Ri, Yo, Hu, Recoverin, Sox1, Titin, Zic4, DNER	opsoclonus myoclonus; cerebellar ataxia; dementia	N/A	N/A	--	SERUM	NEG	NEG	NO
Suspected AIE/PNS #9	2008	**IIFT monospecific** (Oxford): VGKC	limbic encephalitis, epilepsy, catatonia	neuroendocrine tumor (para-trackea)	N/A	--	CSF	**POS** (CSF)	**POS** (CSF)	**YES** (neg)
Suspected AIE/PNS #10	2019	**IIFT primate brain tissue** (Sion)<br>**Immunoblot** (Sion): GAD-65, Amphiphysin, CV2/CRMP5, Ma2, Ri, Yo, Hu, Recoverin, Sox1, Titin, Zic4, DNER/Tr	Cerebellar degeneration	ovarian adenocarcinoma	Prednisone	2 months	SERUM	NEG	NEG	NO

Suspected AIE/PNS #11	2018	**IIFT mouse brain tissue** (Krone)<br>**IIFT monospecific CBA** (Krone): GAD-65, NMDAR, GABABR, AMPAR1, AMPAR2, DPPX, LGI-1, CASPR2, Gly-R, mGluR5<br>**IIFT monospecific CBA** (Geneva): AQP4<br>**Immunoblot** (Krone): Amphiphysin, CV2/CRMP5, Ma2, Ri, Yo, Hu, Recoverin, Sox1, Titin, Zic4, DNER	Stiff person syndrome	neuroendocrine tumor (ileum)	N/A	--	SERUM	**POS** (Serum)	NEG	**YES** (neg)
Suspected AIE/PNS #12	2021	**IIFT primate brain tissue** (Sion)<br>**IIFT monospecific CBA** (Sion): NMDAR, GABABR, AMPAR1, AMPAR2, DPPX, LGI-1, CASPR2, Gly-R, mGluR5, IgLON5, AQP4, MOG<br>**Immunoblot** (Sion): GAD-65, Amphiphysin, CV2/CRMP5, Ma2, Ri, Yo, Hu, Recoverin, Sox1, Titin, Zic4, DNER/Tr	opsoclonus myoclonus	ovarian teratoma	N/A	--	SERUM CSF	NEG	NEG	NO

[a] Times expressed at sampling date
[b] Results from the 96-well hiPSC-derived CNS CBA are expressed as outlier **POS** (positive) or NEG (negative). If POS, in brackets the sample(s) to be tested as an outlier in the corresponding 96-well hiPSC-derived CNS CBA either using neurons or astrocytes.
[c] Samples from patients found to be positive in our novel 96-well hiPSC-derived CNS CBA were evaluated by indirect immunofluorescence on a mouse tissue composite (TIF) (YES or NO). If YES, in brackets the result of the mouse TIF.
[d] Detailed testing information not found in archived medical files. However, note that the patient fulfilled AIE/PNS diagnostic criteria as defined by Graus et al (5, 21)
Abbreviations: AIE : autoimmune encephalitis; PNS : paraneoplastic syndrome; CBA, cell-based assay; IIFT, indirect immunofluorescence test; N/A, not applicable ; CSF, cerebrospinal fluid; neg, negative.

Supplementary Table 7. Individual clinical information of the OIND and NIND patients enrolled in this study (part of exploratory cohort).

Subject	Sampling Year	Diagnosis	Immune modifying treatment[a]	Treatment duration[a]	Tested sample(s)[b]	96-well CBA neuron[b]	96-well CBA astrocyte[b]	Mouse TIF evaluation[c]
OIND #1	2015	Inflammatory temporal lobe mass	N/A	--	SERUM CSF	NEG	**POS** (Serum/CSF)	**YES** (Neg)
OIND #2	2021	Behcet Disease	Prednisone	53 days	SERUM CSF	NEG	NEG	NO
OIND #3	2013	Chronic meningitis (unknow origin)	N/A	--	SERUM	NEG	NEG	NO
OIND #4	2015	Inflammatory cranial neuritis	N/A	--	SERUM	NEG	NEG	NO
OIND #5	2012	Myelitis of unknown origin	N/A	--	CSF	NEG	NEG	NO
OIND #6	2022	Susac syndrome	Rituximab	9.3 months	SERUM	NEG	NEG	NO
OIND #7	2006	Susac syndrome	N/A	--	SERUM CSF	NEG	NEG	NO
OIND #8	2011	Unknown demyelinating disease	N/A	--	SERUM CSF	NEG	NEG	NO
OIND #9	2020	Vasculitis	N/A	--	CSF	NEG	NEG	NO
OIND #10	2018	Vasculitis	N/A	--	SERUM	NEG	NEG	NO
OIND #11	2021	Vasculitis	N/A	--	SERUM CSF	NEG	NEG	NO
OIND #12	2013	Vogt-Koyanagi-Harada syndrome	N/A	--	SERUM CSF	NEG	NEG	NO
OIND #13	2012	Mutlifocal mononeuropathy	N/A	--	SERUM CSF	NEG	NEG	NO
NIND #1	2018	Acrocyanosis	N/A	--	SERUM CSF	NEG	NEG	NO
NIND #2	2011	Acroparesthesia	N/A	--	SERUM CSF	NEG	NEG	NO
NIND #3	2006	Amyotrophic lateral sclerosis	N/A	--	CSF	NEG	**POS** (CSF)	**YES** (Neg)
NIND #4	2013	Amyotrophic lateral sclerosis	N/A	--	SERUM CSF	NEG	NEG	NO

NIND #5	2005	Amyotrophic lateral sclerosis	N/A	--	CSF	NEG	NEG	NO
NIND #6	2006	Amyotrophic lateral sclerosis	N/A	--	CSF	NEG	NEG	NO
NIND #7	2006	Amyotrophic lateral sclerosis	N/A	--	CSF	NEG	NEG	NO
NIND #8	2008	Amyotrophic lateral sclerosis	N/A	--	CSF	NEG	NEG	NO
NIND #9	2019	Catatonia with unknown origin	N/A	--	SERUM CSF	NEG	NEG	NO
NIND #10	2012	Cognitive disorders (memory and attention deficits	N/A	--	SERUM CSF	NEG	NEG	NO
NIND #11	2017	Depression	N/A	--	SERUM CSF	NEG	NEG	NO
NIND #12	2011	Depression	N/A	--	SERUM CSF	NEG	NEG	NO
NIND #13	2020	Depression	N/A	--	SERUM	NEG	NEG	NO
NIND #14	2020	Dysesthesias	N/A	--	SERUM CSF	NEG	NEG	NO
NIND #15	2012	Dysesthesias	N/A	--	SERUM CSF	NEG	NEG	NO
NIND #16	2007	Epilepsy	N/A	--	SERUM CSF	NEG	NEG	NO
NIND #17	2016	Epilepsy	N/A	--	CSF	NEG	NEG	NO
NIND #18	2006	Fibromyalgia	N/A	--	CSF	NEG	NEG	NO
NIND #19	2019	Functional syndrome	N/A	--	SERUM/CSF	NEG	NEG	NO
NIND #20	2021	Genetic myelopathy	N/A	--	SERUM CSF	NEG	NEG	NO
NIND #21	2020	Headache	N/A	--	CSF	NEG	NEG	NO
NIND #22	2020	Headache	N/A	--	CSF	NEG	NEG	NO
NIND #23	2006	Inclusion-body myositis	N/A	--	CSF	NEG	**POS** (CSF)	**YES** (Neg)

NIND #24	2019	idiopathic facial palsy	N/A	--	SERUM CSF	NEG	NEG	NO
NIND #25	2012	Intracranial hypertension	N/A	--	SERUM CSF	NEG	NEG	NO
NIND #26	2013	Intracranial hypertension	N/A	--	SERUM CSF	NEG	NEG	NO
NIND #27	2020	Intracranial hypertension	N/A	--	CSF	NEG	NEG	NO
NIND #28	2020	Ischaemic myelopathy	N/A	--	SERUM CSF	NEG	NEG	NO
NIND #29	2016	Kleine Levin Syndrome	N/A	--	SERUM	NEG	NEG	NO
NIND #30	2012	Migraine	N/A	--	SERUM	NEG	NEG	NO
NIND #31	2016	Migraine	N/A	--	SERUM CSF	NEG	NEG	NO
NIND #32	2017	Migraine	N/A	--	SERUM CSF	NEG	NEG	NO
NIND #33	2018	Migraine	N/A	--	SERUM CSF	NEG	NEG	NO
NIND #34	2018	Migraine	N/A	--	SERUM CSF	NEG	NEG	NO
NIND #35	2020	Migraine	N/A	--	SERUM	NEG	NEG	NO
NIND #36	2013	Mononeuropathy	N/A	--	SERUM CSF	NEG	NEG	NO
NIND #37	2021	Non ischemic cerebral enhancing	N/A	--	SERUM CSF	**POS** (Serum)	NEG	**YES** (Neg)
NIND #38	2021	non inflammatory CNS white matter lesions	N/A	--	SERUM CSF	NEG	NEG	NO
NIND #39	2021	optic chiasma syndrome	N/A	--	SERUM CSF	NEG	NEG	NO
NIND #40	2005	Parkinsonism syndrome	N/A	--	CSF	NEG	NEG	NO
NIND #41	2014	Parkinsonism syndrome	N/A	--	SERUM CSF	NEG	NEG	NO
NIND #42	2014	Parkinsonism syndrome	N/A	--	SERUM CSF	NEG	NEG	NO
NIND #43	2018	Parkinsonism syndrome	N/A	--	SERUM CSF	NEG	NEG	NO

NIND #44	2009	Parkinsonism syndrome	N/A	--	CSF	NEG	NEG	NO
NIND #45	2009	Parkinsonism syndrome	N/A	--	CSF	NEG	NEG	NO
NIND #46	2021	Perinatal periventricular lesion	N/A	--	SERUM CSF	NEG	NEG	NO
NIND #47	2013	Radiculopathy	N/A	--	SERUM CSF	NEG	NEG	NO
NIND #48	2020	Radiculopathy	N/A	--	SERUM CSF	NEG	NEG	NO
NIND #49	2016	Radiculopathy	N/A	--	SERUM CSF	NEG	NEG	NO
NIND #50	2018	Reversible cerebral vasoconstriction syndrome	N/A	--	SERUM CSF	NEG	NEG	NO
NIND #51	2011	Spastic paraparesis	N/A	--	SERUM CSF	NEG	NEG	NO
NIND #52	2013	Spinal stenosis	N/A	--	SERUM CSF	NEG	NEG	NO
NIND #53	2005	Spinal stenosis	N/A	--	CSF	NEG	NEG	NO
NIND #54	2018	Spinal stenosis	N/A	--	CSF	NEG	NEG	NO
NIND #55	2021	Stroke	N/A	--	SERUM CSF	**POS** (Serum)	NEG	NO
NIND #56	2018	Stroke	N/A	--	SERUM CSF	NEG	NEG	NO
NIND #57	2012	Stroke	N/A	--	SERUM CSF	NEG	NEG	NO

[a] Times expressed at sampling date

[b] Results from the 96-well hiPSC-derived CNS CBA are expressed as outlier **POS** (positive) or NEG (negative). If POS, in brackets the sample(s) to be tested as an outlier in the corresponding 96-well hiPSC-derived CNS CBA either using neurons or astrocytes.

[c] Samples from patients found to be positive in our novel 96-well hiPSC-derived CNS CBA were evaluated by indirect immunofluorescence on a mouse tissue composite (TIF) (YES or NO). If YES, in brackets the result of the mouse TIF.

Abbreviations: OIND; other inflammatory neurological disorders; NIND, non-inflammatory neurological disorders; CSF, cerebrospinal fluid; N/A, not applicable.
